# Lesões Plexiformes em Modelo Experimental de Hipertensão Arterial Pulmonar Induzida por Monocrotalina

**DOI:** 10.36660/abc.20190306

**Published:** 2020-09-18

**Authors:** Douglas Mesadri Gewehr, Gabriela Rodrigues Salgueiro, Lucia de Noronha, Fernando Bermudez Kubrusly, Luiz Fernando Kubrusly, Gabriel Antonio Coltro, Paola Cardoso Preto, Andressa de Souza Bertoldi, Heloisa Iacomo Vieira

**Affiliations:** 1 Faculdade Evangélica Mackenzie do Paraná Curitiba PR Brasil Faculdade Evangélica Mackenzie do Paraná (FEMPAR), Curitiba, PR - Brasil; 2 Instituto Denton Cooley de Pesquisa, Ciência e Tecnologia Curitiba PR Brasil Instituto Denton Cooley de Pesquisa, Ciência e Tecnologia (IDC),Curitiba, PR - Brasil; 3 Pontifícia Universidade Católica do Paraná Departamento de Medicina Curitiba PR Brasil Pontifícia Universidade Católica do Paraná Departamento de Medicina, Curitiba, PR - Brasil; 4 Instituto do Coração de Curitiba Curitiba PR Brasil Instituto do Coração de Curitiba (InCor Curitiba), Curitiba, PR - Brasil; 5 Centro de Estudos e Pesquisa em Emergências Médicas e Terapia Intensiva Curitiba PR Brasil Centro de Estudos e Pesquisa em Emergências Médicas e Terapia Intensiva (CEPETI), Curitiba, PR - Brasil; 6 Hospital Universitário Evangélico Mackenzie Curitiba PR Brasil Hospital Universitário Evangélico Mackenzie (HUEM), Curitiba, PR – Brasil

**Keywords:** Monocrotalina, Remodelação Vascular, Neointima, Hipertensão Pulmonar, Hipertrofia Ventricular Direita

## Abstract

**Fundamento:**

O modelo de hipertensão arterial pulmonar induzida por monocrotalina (MCT) é um dos mais reproduzidos atualmente, apresentando como limitação a ausência de lesões plexiformes, manifestações típicas da doença grave em humanos.

**Objetivo:**

Avaliar a gravidade da arteriopatia pulmonar induzida por MCT por meio dos achados anatomopatológicos pulmonares e cardíacos, evolução clínica e sobrevida em 37 dias.

**Métodos:**

Foram utilizados 50 ratos machos Wistar divididos em quatro grupos, sendo um controle (n = 10). Os três grupos restantes foram submetidos à inoculação de MCT (60 mg/kg i.p.) e ficaram sob o seu efeito por 15 (n = 10), 30 (n = 10) e 37 dias (n = 20). Ao final de cada período, os animais foram sacrificados, obtendo-se tecidos pulmonar e cardíaco para análise anatomopatológica e morfométrica. Empregou-se o teste Kruskal-Wallis, considerando nível de significância de 5%.

**Resultados:**

Nos pulmões dos animais MCT foram constatadas lesões referentes à arteriopatia pulmonar, incluindo muscularização das arteríolas, hipertrofia da camada média e lesões neointimais concêntricas. Lesões complexas foram observadas nos grupos MCT, descritas como plexiforme e do “tipo” plexiforme (plexiform-like). A hipertrofia do ventrículo direito foi constatada pelo aumento da espessura e diâmetro dos cardiomiócitos e pelo aumento significativo da espessura da parede do ventrículo direito (p<0,0000).

**Conclusão:**

O modelo foi capaz de gerar arteriopatia pulmonar moderada-grave associada à hipertrofia do ventrículo direito secundária, com sobrevida de 50% em 37 dias. De nosso conhecimento, este estudo foi o primeiro a constatar a presença de lesões vasculares complexas, semelhantes às observadas em pacientes com hipertensão arterial pulmonar grave, em modelo isolado de MCT. (Arq Bras Cardiol. 2020; 115(3):480-490)

## Introdução

A hipertensão arterial pulmonar (HAP) é uma situação clínica de elevada gravidade, caracterizada pela presença de vasoconstrição pulmonar, trombose *in situ* e remodelamento vascular.^1^O aumento progressivo da resistência vascular pulmonar (RVP) resulta em uma hipertrofia compensatória do ventrículo direito (VD), progredindo para insuficiência cardíaca e morte prematura.^2^

Na HAP avançada, a proliferação endotelial e a hipertrofia do músculo liso vascular resultam em obstrução do lúmen arterial. Na maioria dos casos, ocorre a formação de lesões neointimais concêntricas e lesões hipercelulares complexas, conhecidas como lesões plexiformes.^3^As alterações anatomopatológicas da HAP foram primeiramente caracterizadas por Heath & Edwards^4^ em 1958. Posteriormente, Wagenvoort & Wagenvoort^5^ em 1977 descreveram a sequência de alterações vasculares na HAP, definindo-a como Arteriopatia Pulmonar Plexogênica (APP). Essas alterações parecem refletir, em geral, o nível pressórico da artéria pulmonar e, em menor grau, o tempo de HAP.^6^

Como o tecido humano com doença em estágio inicial raramente está disponível, um modelo animal que reproduza as alterações precoces da arteriopatia pulmonar e sua evolução seria desejável para compreender a complexidade da HAP e encontrar novas estratégias terapêuticas.^7^

O modelo animal de HAP induzida por monocrotalina (MCT) é um dos mais realizados pelos pesquisadores, pois oferece simplicidade técnica, fácil reprodutibilidade e baixo custo em comparação com os outros modelos.^7^ Além disso, é capaz de mimetizar vários aspectos-chave da HAP humana, incluindo remodelamento vascular, proliferação de células musculares lisas, disfunção endotelial, aumento da expressão de citocinas inflamatórias e falência do ventrículo direito.^1,8,9^ Tipicamente, esse modelo é baseado em uma única injeção de MCT (geralmente 60 mg/kg) aplicada por via intraperitoneal (i.p.) ou subcutânea, resultando no desenvolvimento de HAP após em 3-4 semanas.^7,10^

A MCT é um alcaloide pirrolizidínico presente nos caules, folhas e sementes de plantas do gênero *Crotalaria sp.* (principalmente as espécies *spectabilis, retusa* e *sigma*), leguminosas distribuídas principalmente nas regiões de clima tropical.^10^ A monocrotalina, ao ser metabolizada no fígado pelo citocromo p-450, é transformada em sua forma ativa, a monocrotalina pirrole, exercendo sua toxicidade no sistema cardiopulmonar.^7,10^ Seu uso foi primeiramente descrito há mais de 50 anos por Kay et al.,^11^ primeiros a reportarem HAP causada pela ingestão de sementes de *Crotalaria spectabilis* em ratos. Relatos anteriores haviam descrito arterite pulmonar em ratos alimentados com sementes da planta^12^ e identificaram o alcaloide pirrolizidínico como o agente causador.^13^

Embora muitas evidências sugiram que a MCT induz disfunção endotelial das arteríolas pulmonares em múltiplos níveis, o modelo de HAP por MCT é caracterizado predominantemente por hipertrofia da camada média. A ausência de lesões complexas, constatadas na HAP moderada-grave, apresenta-se como uma importante limitação desse modelo.^7,14-16^

A proposta do presente estudo consiste em reproduzir o modelo experimental de HAP induzida por MCT em ratos Wistar com o objetivo de avaliar a gravidade da arteriopatia pulmonar por meio dos achados anatomopatológicos pulmonares, repercussões cardíacas, evolução clínica e sobrevida em 37 dias.

## Métodos

No presente estudo experimental foram respeitadas as normas estabelecidas no “Guide for the Care and Use of Laboratory Animals” e os Princípios Éticos na Experimentação Animal do Conselho Nacional do Controle de Experimentação Animal (CONCEA). O estudo teve aprovação do Comissão de Ética no Uso de Animais da Faculdade Evangélica Mackenzie do Paraná (CEUA/FEMPAR), registrado sob o protocolo n. 3433/2016.

Cinquenta ratos machos Wistar, da espécie *Rattus norvegicus*, pesando entre 250 e 300 gramas, foram distribuídos aleatoriamente em quatro grupos (randomização simples): grupo controle (GC) (n=10) – animais que receberam uma injeção intraperitoneal de solução fisiológica (0,9%, 1 mL/kg) no dia de início do experimento (D0); grupos MCT – animais que receberam uma injeção intraperitoneal de MCT (60 mg/kg)^17^(Sigma-Aldrich, St. Louis, MO, EUA), dissolvida em solução fisiológica (0,9%) no D0. Os animais ficaram sob o efeito da MCT por 15 dias, 30 dias e 37 dias, representando os grupos G15 (n=10), G30 (n=10), G37 (n=20), respectivamente.

Nos dias 15 (G15), 30 (G30) e 37 (G37 e GC) do experimento, após a anestesia com uma combinação de 0,3 mg/kg de cloridrato de xilazina 2% (Xilazin^®^; Syntec, São Paulo, Brasil) e 10 mg/kg de cloridrato de cetamina 10% (Cetamin^®^; Syntec, São Paulo, Brasil) i.p.,^17^ os animais foram pesados e posteriormente sacrificados por meio de exsanguinação por punção cardíaca. Por fim, foi conduzida a retirada de ambos os pulmões e do coração, os quais foram pesados através de balança semi-analítica de precisão (AD200; © Marte Científica, São Paulo, Brasil).

Os órgãos coletados foram processados segundo técnica histológica convencional. Para cada animal foram confeccionadas duas lâminas histológicas de tecido pulmonar, uma com três cortes transversais contendo os lobos pulmonares direitos, e a outra com um corte transversal e um longitudinal contendo os lobos pulmonares esquerdos. As lâminas de pulmão foram coradas com hematoxilina-eosina (HE), tricrômico de Mallory e coloração de Weigert para fibras elásticas.^14^As lâminas de coração incluíram duas secções transversais de tecido cardíaco, um ao nível do terço médio dos ventrículos e outro ao nível dos vasos da base, corados com HE.

As lâminas de pulmão foram avaliadas histopatologicamente utilizando um método de pontuação semiquantitativo (0 – sem alteração; 1 – discreto; 2 – moderado/acentuado) de parâmetros de remodelação vascular e alterações parenquimatosas, descritas a seguir: espessamento das paredes alveolares, edema intersticial, exsudato alveolar, hipertrofia da camada média, infiltrado leucocitário e proliferação intimal/neointimal.

As lâminas de tecido pulmonar e cardíaco foram digitalizadas com um scanner (Axio Scan Z1, Zeiss, Jena, Alemanha) (40X) e as imagens foram analisadas pelo software ZEN 2.3 (blue edition) (© Carl Zeiss Microscopy GmbH, 2011), que permite medidas quantitativas geométricas. A quantificação histológica da espessura da parede do ventrículo direito (EPVD) foi realizada em um aumento de 10X, expressa em micrômetros (µm). A região central da parede do VD foi definida como padrão para a análise desse parâmetro em todas as lâminas. A quantificação da área da câmara ventricular direita foi realizada em um aumento de 10X e expressa em micrômetros quadrados (µm^2^).

### Análise Estatística

As variáveis morfométricas (contínuas) foram representadas por gráficos *boxplot*, que expressam os dados em mediana e intervalo interquartil. A suposição de normalidade das variáveis foi avaliada pelo teste de Kolmogorov-Smirnov. As variáveis não apresentaram distribuição normal e, por este motivo, foram analisadas e comparadas com o teste não paramétrico de Kruskal-Wallis, usando o método de Simes e Hochberg para múltiplas comparações.

O tamanho amostral e sua distribuição nas parcelas experimentais basearam-se na experiência em trabalhar com estes animais nestas condições, na viabilidade estatística do desenho experimental, nos dados descritos na literatura médica e nas normas estabelecidas pelo CONCEA e CEUA/FEMPAR. Utilizando o teste de Kolmogorov-Smirnov na comparação entre os grupos, mostrou-se necessário um número de oito animais por grupo experimental. Levando em conta a mortalidade de 20% por grupo devido a problemas de procedimento, e de 50% a partir da quarta semana devido à toxicidade da MCT, necessitou-se mais dois animais no GC, G15 e G30 e doze no grupo G37, totalizando uma amostra de 50 animais.

Os valores de p abaixo de 0,05 foram considerados estatisticamente significantes. Os dados foram analisados com o software estatístico Action Stat (versão 3.5.152.34).

## Resultados

### Evolução e Mortalidade

Com o passar das semanas, foi possível observar o aparecimento gradativo de sinais de doença pulmonar nos animais dos grupos MCT, como anorexia, perda de peso, irregularidade do padrão respiratório, intolerância aos esforços e cianose de extremidades. Esses sinais se tornaram mais evidentes nos animais do G30 e G37, que se apresentavam mais irritados e com espirros frequentes. No GC, G15 e G30 todos os animais sobreviveram. Contudo, no G37, houve uma mortalidade de 50%.

### Peso Corpóreo e Peso Relativo dos Órgãos

**Figura 1:**
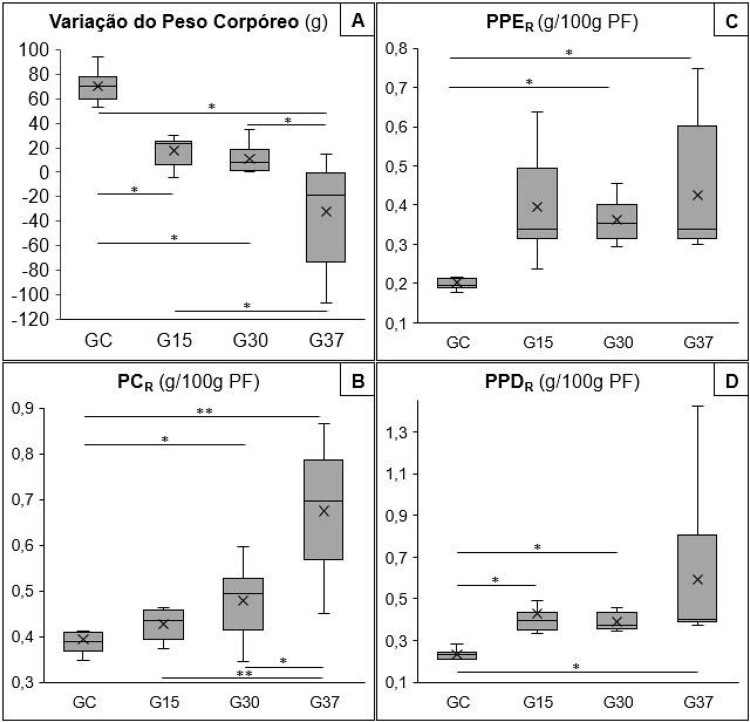
– Variação do peso corpóreo e peso relativo dos órgãos. Comparação entre os animais dos grupos controle (GC), 15 dias (G15), 30 dias (G30) e 37 dias (G37), ao final do período experimental. Em A, variação de peso corpóreo, em gramas, *p ≤ 0,0001; em B, peso relativo cardíaco (PCR), em g/100g de peso final (PF), *p < 0,005 e **p ≤ 0,0000; em C e D, peso relativo pulmonar esquerdo e direito (PPER e PPDR), em g/100g de peso final, *p ≤ 0,0000

Na Figura 1A, observa-se uma diminuição do ganho de peso à medida que os animais ficaram mais tempo expostos aos efeitos da MCT, de modo que os animais do G37 tiveram uma redução significativa, de cerca de 11%, do seu peso final em relação ao inicial, em comparação ao GC e G30 (p<0,0001).

O peso relativo dos pulmões dos animais foi significativamente maior nos grupos MCT em relação ao GC (p<0,0000), como se observa nas figuras 1C e 1D. Entretanto, esse aumento não teve um padrão linear, apresentando uma leve queda no G30. O peso relativo dos corações foi significativamente maior nos grupos G30 e G37 (p<0,005) em relação ao GC (Figura 1B).

### Achados Macroscópicos

Os pulmões dos animais dos grupos G15 e G30 apresentavam-se macroscopicamente aumentados de volume, alguns sem achados de superfície, outros levemente hiperemiados e congestos. A maioria dos pulmões dos animais do G37 apresentavam intensa congestão pulmonar, 30% deles com petéquias e 20% deles apresentavam aspecto ferruginoso. O acometimento pulmonar direito foi mais intenso do que o esquerdo, a nível macroscópico. No dia da morte, em metade dos animais do G37, foi observado líquido sero-hialino em cavidades pleurais e líquido sero-hemático em cavidade pericárdica, além de ascite e congestão hepática. Os demais órgãos não apresentavam alterações macroscópicas visíveis, com exceção do fígado, que apresentava-se congesto e com aspecto de noz-moscada.

### Achados Histopatológicos Pulmonares

**Figura 2:**
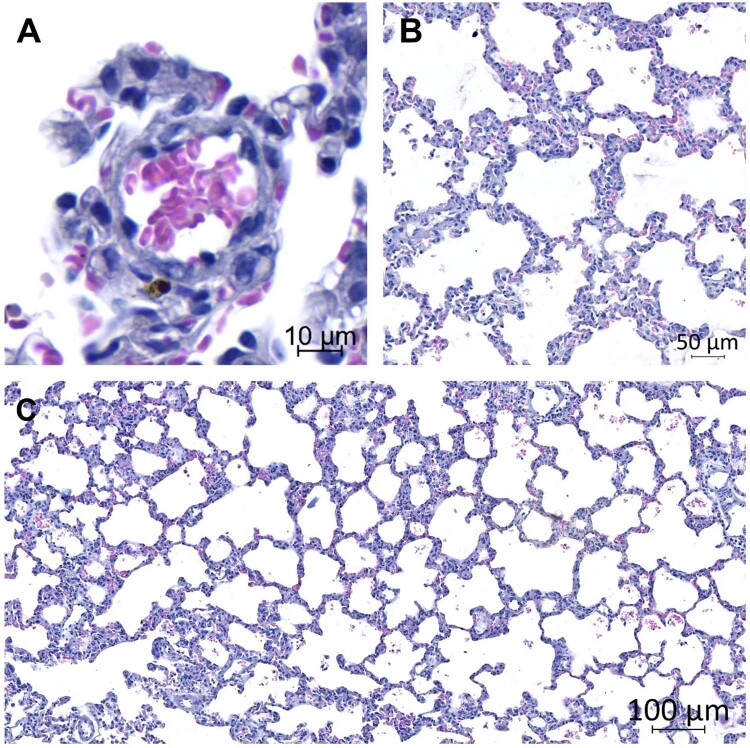
– Fotomicrografias de tecido pulmonar do grupo controle. Observam-se alvéolos íntegros ocupando a maior parte do parênquima pulmonar (B e C) e arteríolas com estruturas e dimensões normais (A). Coloração hematoxilina-eosina; objetivas de 20X (A), 5X (B) e 2X (C)

**Figura 3:**
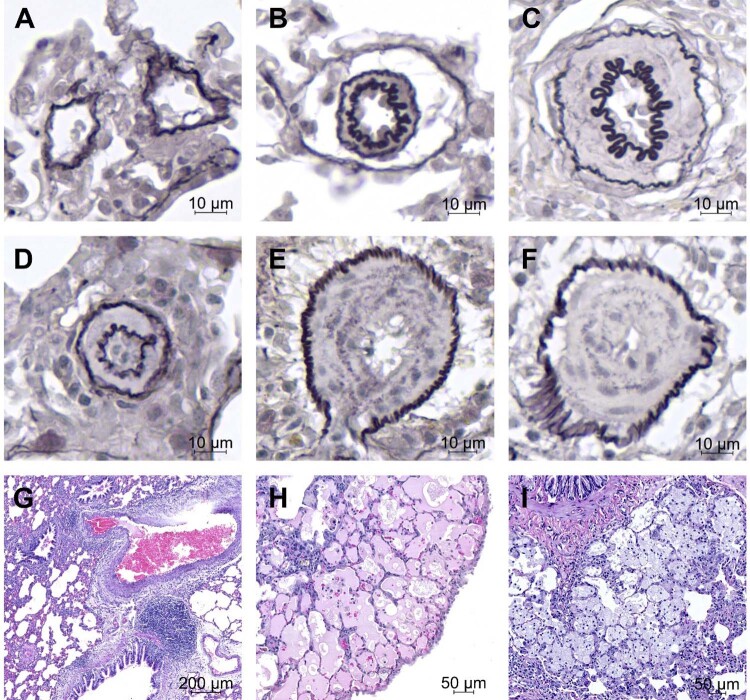
– Achados histopatológicos vasculares e parenquimatosos do tecido pulmonar. Fotomicrografias de cortes histológicos do tecido pulmonar dos animais do grupo controle (GC) e de animais que receberam monocrotalina (grupos MCT); A – F, avaliação da remodelação arterial pulmonar observadas nas arteríolas alveolares vistas em cortes transversais (coloração de Weigert; objetiva 20X); G – I, alterações parenquimatosas nos animais dos grupos MCT (coloração hematoxilina-eosina; objetiva 1X, 5X, 5X, respectivamente); A e B, arteríolas alveolares do GC com estruturas e dimensões normais; C – F, arteríolas dos grupos 15, 30 e 37 dias; C e D, muscularização e hipertrofia da camada média, com proliferação neointimal concêntrica (D); E e F, lesões neointimais laminares celulares concêntricas com intensa redução do lúmen vascular e ruptura da membrana elástica externa (E); G, infiltrado inflamatório no interstício hilar, peribronquiolar e perivascular. Parênquima pulmonar apresenta poucas áreas alveolares aeradas associada a uma intensa exsudação intra-alveolar do tipo serofibrinosa (H) e macrofágica (I), com a presença de células esponjosas

Nas fotomicrografias de tecido pulmonar dos animais do GC, observou-se alvéolos íntegros ocupando a maior parte do parênquima pulmonar, adequadamente aerados (Figuras 2B e 2C), e arteríolas com estrutura e dimensões normais (Figuras 2A, 3A, 3B e 4A).

**Figura 4:**
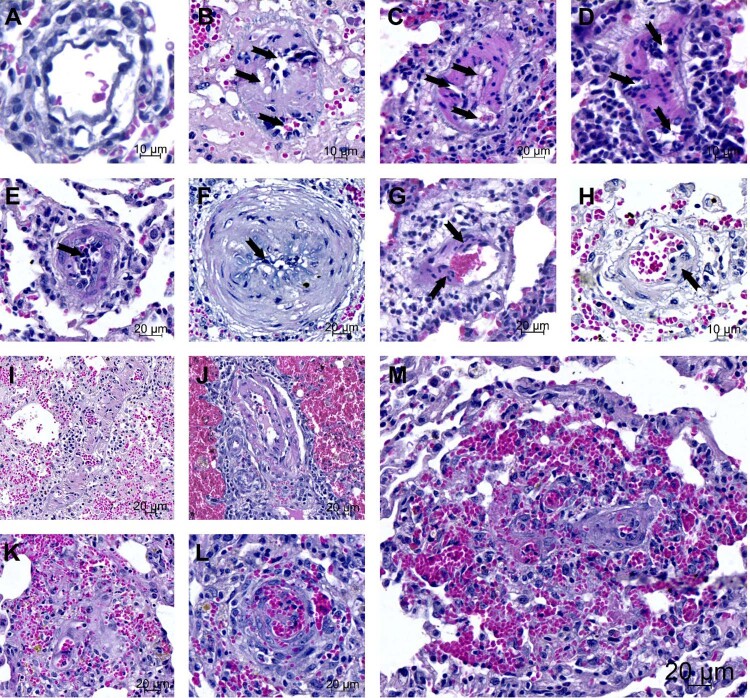
– Lesões vasculares complexas do tecido pulmonar. Fotomicrografias representativas de lesões complexas do tecido pulmonar dos animais que receberam monocrotalina (grupos MCT) (coloração hematoxilina-eosina; objetiva 10X); A, arteríola alveolar do grupo controle com estrutura e dimensões normais; B – H, lesões “plexiform-like” (“stalk-like lesions”) em cortes transversais; B – D, arteríolas apresentando canais parecidos com fendas (“slit-like channels”), indicados pelas setas. E – H, arteríolas com pequenas massas de células hipercromáticas semelhantes a um broto (“bud-like”), projetando-se em direção ao lúmen vascular, indicadas pelas setas; I – M, lesões plexiformes em cortes transversais (grupo do dia 37, G37) mostrando a combinação de múltiplos pequenos canais capilares e pequenas fendas

A análise histológica dos animais MCT revelou alterações parenquimatosas pulmonares, como exsudato alveolar, espessamento das paredes alveolares, edema intersticial e infiltrado leucocitário (Figura 3G). Foram encontrados três tipos de exsudatos ocupando a luz alveolar: serofibrinoso (Fig. 3H), hemorrágico e macrofágico (Fig. 3I). As artérias e arteríolas pulmonares apresentavam várias formas de remodelação vascular, incluindo muscularização e hipertrofia da camada média (Figuras 3C e 3D), proliferação intimal, vários graus de espessamento neointimal, concêntrico (Figura 3D) e laminar concêntrico (Figuras 3E e 3F), e lesões hipercelulares complexas.

No processo de muscularização, a arteríola adquiriu uma dupla membrana elástica com um novo músculo entre elas. A proliferação das células da íntima levou a uma íntima espessada, sem nenhuma organização especial, afetando toda a circunferência do vaso.

Foram observados dois padrões diferentes de lesões hipercelulares complexas em relação a sua morfologia. Primeiro, lesões semelhantes a pedúnculos (“stalk-like/plexiforme-like lesions”), formadas no lúmen da arteríola pulmonar (Figuras 4B-H). O corpo da lesão apresenta-se como um massa desordenada semelhante a um pedúnculo, formada por células hipercromáticas e ovais, que parecem surgir da parede arterial, estendendo-se a jusante no lúmen do vaso. As secções transversais mostraram arteríolas com muitos canais parecidos com fendas (“slit-like channels”) separados por células centrais hipercromáticas (Figuras 4B-D). Ainda, foram observadas pequenas massas de células semelhantes a um broto (“bud-like”), projetando-se da parede arterial para o lúmen (Figuras 4E-H).

O segundo padrão encontrado foram lesões plexiformes (Figuras 4I-M), mostrando arteríolas frequentemente dilatadas com lúmen caracteristicamente preenchido por canais capilares e “slit-like channels”. Essas lesões foram observadas quase que exclusivamente no G37, sem acometer todos os animais do grupo, apresentando distribuição focal no tecido pulmonar.

Nos grupos G30 e G37 houve uma intensificação das injúrias vasculares e parenquimatosas, com destaque na hipertrofia da camada média, proliferação intimal e exsudato alveolar. Entretanto, o G37 mostrou uma diminuição no infiltrado leucocitário em comparação com o G15 e G30.

**Figura 5:**
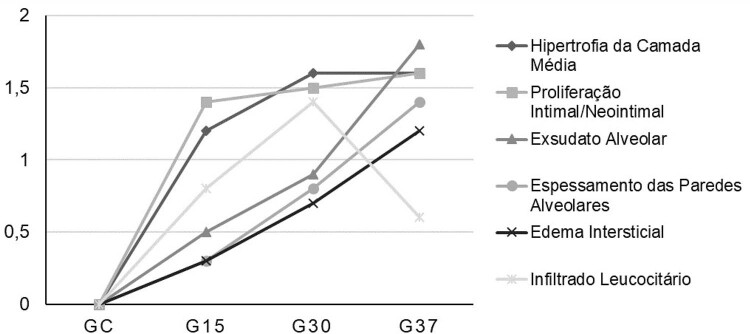
– Evolução dos Parâmetros de Remodelação Vascular e Alterações Parenquimatosas. Avaliação Semiquantitativa (0 – sem alteração; 1 – discreto; 2 – moderado/acentuado)

Os resultados da avaliação semiquantitativa da vasculatura e parênquima pulmonar encontram-se esquematizados na Figura 5.

### Achados Histopatológicos e Morfométricos Cardíacos

**Figura 6:**
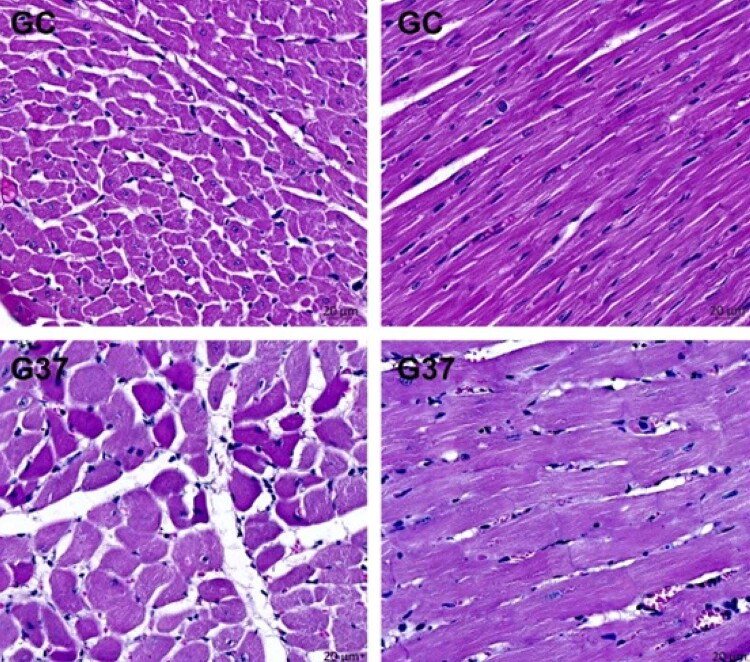
– Achados histopatológicos do tecido cardíaco. Fotomicrografias de corte transversal (à esquerda) e longitudinal (à direita) do ventrículo direito de animais dos grupos controle (GC) e grupo 37 dias (G37) (coloração hematoxilina-eosina; objetiva 20X)

O músculo cardíaco também sofreu alterações anatomopatológicas expressivas. A Figura 6 mostra fotomicrografias de corte transversal e longitudinal do ventrículo direito dos animais do GC e G37. Observam-se núcleos íntegros dispostos perifericamente e feixes de fibras íntegras, porém com aumento de espessura e diâmetro no G37.

**Figura 7:**
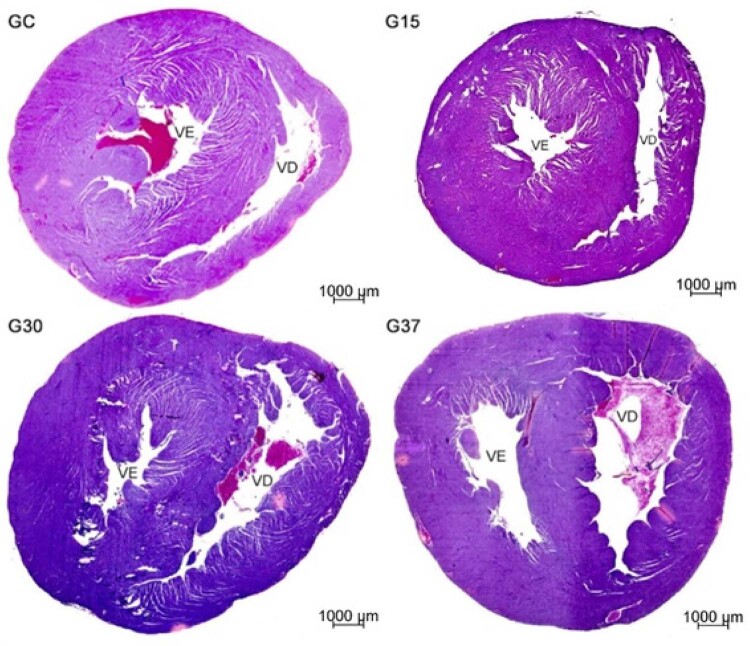
– Evolução da hipertrofia e dilatação do ventrículo direito. Fotomicrografias de corte transversal ao nível do 1/3 médio dos ventrículos cardíacos de animais do grupo controle (GC), 15 dias (G15), 30 dias (G30) e 37 dias (G37) (coloração hematoxilina-eosina)

**Figura 8:**
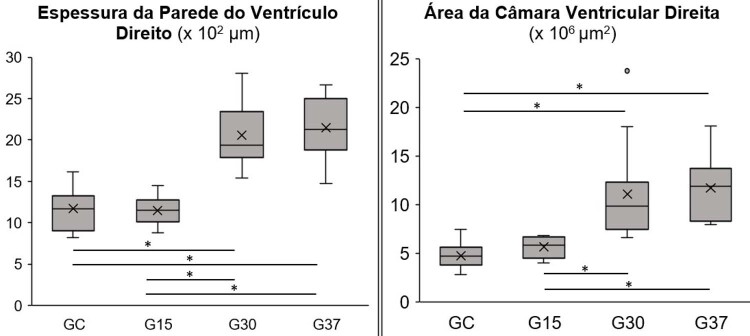
– Comparação da espessura da parede do ventrículo direito e da área da câmara ventricular direita entre os grupos. Comparação da espessura da parede do ventrículo direito e da área da câmara ventricular direita entre os animais do grupo controle (GC), grupo 15 dias (G15), grupo 30 dias (G30) e grupo 37 dias (G37); *p < 0,0000

A hipertrofia ventricular direita, evidentemente retratada na Figura 7, foi quantificada pela medida da EPVD e sua variação entre os grupos (Figura 8). Não houve diferença significativa na medida da EPVD entre os animais do GC e G15. A medida da EPVD aumentou significativamente no G30/G37 (p<0,0000), representando quase duas vezes o valor do GC.

Outro importante achado foi a dilatação significativa da câmara ventricular direita (Figura 7) dos animais do G30 e G37, quantificada pela medida da área da câmara ventricular direita (p<0,0000) (Figura 8).

## Discussão

Esse é o primeiro relato em que a administração intraperitoneal de MCT (60 mg/kg) foi capaz de deflagrar APP em ratos.

Sinais de doença pulmonar manifestaram-se gradativamente nos animais dos grupos MCT, como já constatado por diversos pesquisadores que reproduziram o modelo.^9,18,19^ A dispneia e a perda de peso são, na maioria das vezes, os primeiros sinais de doença pulmonar. A alteração do pH sanguíneo secundária à ineficiência do pulmão em manter uma correta relação ventilação/perfusão (que explica a cianose de extremidades), contribui para a anorexia e baixo ganho de peso. Nos animais do presente estudo, a cianose de extremidades também pode ser secundária à vasoconstrição periférica e ao baixo débito cardíaco. O esforço respiratório (dispneia) contribui para o baixo ganho de peso (ou perda de peso) à medida que aumenta o gasto metabólico. De fato, o sinal mais comum da HAP é a dispneia e a fadiga, decorrentes do baixo débito cardíaco, de carácter progressivo e indicativo de insuficiência ventricular direita secundária.^20,21^

O aumento do peso relativo pulmonar e o aumento do peso relativo cardíaco dos animais dos grupos MCT são achados já consolidados na literatura.^9,17,18,22^O aumento do peso relativo pulmonar deve-se ao processo de congestão, no qual há uma redução do volume alveolar e um aumento da área ocupada por estruturas extra-alveolares, representadas principalmente pelo exsudato alveolar, espessamento das paredes alveolares e edema intersticial. De maneira análoga, sugere-se que o aumento do PC_R_, é decorrente do aumento da massa ventricular direita hipertrofiada dos animais MCT, fato discutido mais adiante.

Com apenas 15 dias, a forma ativa da MCT foi capaz de deflagrar alterações na vasculatura e no parênquima pulmonar, típicas de doença hipertensiva arterial pulmonar, corroborando com os estudos de Martins.^9^Já outros autores constataram que mudanças significativas nas pressões da artéria pulmonar, hipertrofia da camada média das arteríolas pulmonares e hipertrofia ventricular direita, manifestam-se apenas 3-4 semanas após a administração da MCT.^18, 23,24^

O endotélio ocupa um papel importante na etiopatogênese da HAP, participando de forma expressiva nas alterações do tônus e remodelamento vascular, além do seu envolvimento no processo vaso-oclusivo. Embora exista uma vasta evidência na literatura que sugira a correlação entre MCT e disfunção da barreira endotelial em múltiplos níveis,^8,25,26^ de acordo com muitos estudos, esse modelo é caracterizado predominantemente por hipertrofia da camada média das arteríolas pulmonares e não por proliferação endotelial excessiva, a qual está presente na doença humana.^3,17,27^Tais considerações estão em desacordo com nossa análise histológica do tecido pulmonar, pois tanto a hipertrofia da camada média quanto a proliferação endotelial ocuparam papel expressivo no processo patológico da arteriopatia pulmonar dos animais MCT, dando origem a lesões vasculares complexas, referidas como plexiforme e do “tipo” plexiforme (*plexiform-like*).

Embora as lesões plexiformes observadas na HAP grave humana sejam difíceis de serem reproduzidas em modelos animais, pela primeira vez, um modelo isolado de MCT mostrou a presença dessas lesões. Em particular, caracterizam-se por uma estrutura desorganizada, de aspecto glomerulóide, formada por células hipercromáticas e ovais.^3-5^Essas características, apesar de únicas, permanecem incertas, presumivelmente porque a lesão apresenta um caráter dinâmico e, mais importante, não se sabe como são formadas. Consensualmente, consiste em uma lesão angioproliferativa complexa, composta de canais revestidos por células endoteliais e separados por células centrais.^28,29^Estas lesões complexas, descritas em nosso estudo, apresentam certa semelhança com a morfologia das lesões plexiformes na HAP humana grave.

Como sugerido recentemente por Tuder^28^ em sua revisão da arteriopatia da HAP em humanos, as lesões denominadas “plexiform-like” podem representar um estágio inicial do padrão intraluminal da lesão plexiforme. Como o nome sugere, a complexidade dessas lesões se assemelha à das plexiformes típicas, porém apresentam uma morfologia distinta. Tais lesões, observadas em nosso estudo, são formadas dentro do lúmen vascular, de modo que o corpo da lesão assemelha-se a uma massa desordenada semelhante a um pedúnculo (“stalk-like”), constituída por células hipercromáticas e ovais, que parece surgir da parede arterial e se estende a jusante no lúmen do vaso, adquirindo canais parecidos com fendas (“slit-like channels”), bem como estruturas semelhantes a brotos (“bud-like”).^30^

Vários modelos combinados de HAP, descritos na literatura médica, demonstraram que alterações nas condições hemodinâmicas e/ou no grau de saturação de oxigênio em animais submetidos à inoculação de substâncias tóxicas são capazes de intensificar o seu efeito sobre os vasos pulmonares e, dessa forma, desenvolver lesões vasculares complexas. White et al.,^31^ relataram HAP grave em ratos jovens pneumonectomizados submetidos à inoculação de MCT. Coste et al.,^32^e Morimatsu et al.,^33^ demonstraram o agravamento da HAP por MCT em ratos submetidos à um regime de hipóxia crônica. Ainda, Abe et al.,^30^ demonstraram o desenvolvimento do HAP e arteriopatia pulmonar severa por meio da associação do bloqueador do receptor do fator de crescimento vascular Sugen 5416 à hipóxia crônica. As lesões neointimais laminares e não-laminares concêntricas, observadas em nosso estudo, assemelham-se àquelas encontradas na HAP humana grave^34^ e nos modelos experimentais de HAP mencionados acima.

Wagenvoort & Wagenvoort^5,35^ foram os primeiros a descrever a sequência das alterações vasculares da HAP e um dos pioneiros em avaliar o grau de reversibilidade dessas lesões, estudos válidos até os dias de hoje. Em nosso estudo, os processos de muscularização, hipertrofia da camada média e proliferação intimal/neointimal caracterizaram as alterações iniciais, sendo, portanto, potencialmente reversíveis sob o ponto de vista anatomopatológico. Contudo, as lesões vasculares complexas, mais presentes no G37, podem ser consideradas alterações geralmente irreversíveis, resultando em repercussões hemodinâmicas mais graves e mortalidade precoce, assim como ocorrido.^31^

A constatação das lesões plexiformes em nosso estudo, pode estar relacionada com o tempo de observação prolongado de 37 dias e à maior amplitude da análise anatomopatológica pulmonar. O surgimento das lesões vasculares complexas em HAP é tempo-dependente, ou seja, quanto maior o tempo dos animais expostos à MCT, maior a chance de evolução para as lesões mais complexas. Além disso, por serem focais, a observação das lesões plexiformes no parênquima pulmonar está na dependência da amplitude da análise histológica.

Com a evolução da HAP, o aumento da resistência vascular pulmonar causa uma hipertrofia ventricular direita secundária, em razão do aumento da sobrecarga do VD.^36^Com 30 dias sob o efeito da MCT, os animais do nosso estudo já apresentavam hipertrofia ventricular direita.^9,36,37^A hipertrofia ventricular miocárdica, por não implicar em um aumento da capacidade de contração cardíaca, evolui com dilatação das câmaras direitas e *Cor pumonale*.^9,17^ Sinais de insuficiência cardíaca como derrame pleural, ascite e congestão hepática foram constatados nos animais do G37, assim como em alguns estudos previamente citados.

A espessura da parede do ventrículo direito foi utilizada como marcador de hipertrofia ventricular direita, embora muitos estudos utilizem a razão entre o peso do VD sobre o peso do conjunto ventrículo esquerdo + septo interventricular.^17^ De qualquer forma, ambos os métodos permitiram concluir o desenvolvimento de uma expressiva hipertrofia do VD nos animais MCT. Ainda, a hipertrofia do VD foi evidenciada pelo aumento da espessura e diâmetro dos cardiomiócitos, com preservação da integridade de suas fibras, como constatado por Martins,^9^ Cabrini,^18^ Nogueira- Ferreira et al.,^38^ e Pacagnelli et al.,^39^ E, por fim, a dilatação progressiva das câmaras direitas corrobora os resultados obtidos por Martins,^9^ que descreve dimensões muito superiores às correspondentes do lado esquerdo.

Apesar das semelhanças, esse modelo não mimetiza de maneira fidedigna a HAP em seres humanos. Isso se deve principalmente pelo fato de que não se sabe como as lesões angioproliferativas complexas são formadas e se elas realmente reproduzem os aspectos fisiopatológicos das lesões humanas.

## Conclusão

O experimento demonstrou que uma dose intraperitoneal de 60 mg/kg de MCT foi capaz de gerar arteriopatia pulmonar moderada-grave, com muscularização das arteríolas, hipertrofia da camada média e formação neointimal. O modelo reproduziu múltiplas alterações estruturais no parênquima e nas arteríolas pulmonares, bem como hipertrofia ventricular direita secundária ao aumento da resistência vascular pulmonar. De nosso conhecimento, esse estudo foi o primeiro a constatar a presença de lesões complexas, principalmente as plexiformes, semelhantes às observadas em pacientes com HAP grave em um modelo isolado de MCT.
