# Supplementary material for: Lesões Plexiformes em Modelo Experimental de Hipertensão Arterial Pulmonar Induzida por Monocrotalina
Source: Arq Bras Cardiol. 2020 Sep 18;115(3):480–90. [Article in Portuguese] doi: 10.36660/abc.20190306 (PMC9363102; doi:10.36660/abc.20190306)
Supplement: Translation

# Translation: Plexiform Lesions in an Experimental Model of Monocrotalin-Induced Pulmonary Arterial Hypertension

**Authors:** Douglas Mesadri Gewehr, Gabriela Rodrigues Salgueiro, Lucia de Noronha, Fernando Bermudez Kubrusly, Luiz Fernando Kubrusly, Gabriel Antonio Coltro, Paola Cardoso Preto, Andressa de Souza Bertoldi, Heloisa Iacomo Vieira

**Affiliations:** 1 Brazil Faculdade Evangélica Mackenzie do Paraná (FEMPAR), Curitiba, PR - Brazil; 2 Brazil Instituto Denton Cooley de Pesquisa, Ciência e Tecnologia (IDC), Curitiba, PR - Brazil; 3 Brazil Pontifícia Universidade Católica do Paraná Departamento de Medicina, Curitiba, PR - Brazil; 4 Brazil Instituto do Coração de Curitiba (InCor Curitiba), Curitiba, PR - Brazil; 5 Brazil Centro de Estudos e Pesquisa em Emergências Médicas e Terapia Intensiva (CEPETI), Curitiba, PR - Brazil; 6 Brazil Hospital Universitário Evangélico Mackenzie (HUEM), Curitiba, PR – Brazil

**Keywords:** Monocrotaline, Vascular Remodeling, Neointimal, Pulmonary hypertension, Right Ventricular Hypertrophy

## Abstract

**Background:**

The monocrotaline (MCT)-induced pulmonary arterial hypertension model is one of the most reproduced today, presenting as a limitation the absence of plexiform lesions, typical manifestations of the severe disease in humans.

**Objective:**

To evaluate the severity of MCT-induced pulmonary arteriopathy by pathological findings of lung and heart tissue samples, clinical course and 37-day survival.

**Methods:**

Fifty male Wistar rats were divided into one of the four groups – control (CG) (n = 10) and three intervention (MCT) groups. The MCT groups received intraperitoneal injection (60 mg/kg) of MCT and remained exposed to the substance for 15 days (G15, n = 10), 30 days (G30, n = 10) and 37 days (G37, n = 20). At the end of each period, the animals were sacrificed, and pulmonary and cardiac tissues were collected for anatomopathological and morphometric analysis. The Kruskal-Wallis test was used, considering a level of significance of 5%.

**Results:**

In the lungs of MCT animals, lesions related to pulmonary arteriopathy were found, including muscularization of the arterioles, hypertrophy of the middle layer and concentric neointimal lesions. Complex lesions were observed in MCT groups, described as plexiform and plexiform-like lesions. Right ventricular hypertrophy was evidenced by increased thickness and diameter of the cardiomyocytes and a significant increase in the right ventricular wall thickness (p <0.0000).

**Conclusion:**

The MCT model was able to generate moderate-severe pulmonary arteriopathy associated with secondary right ventricular hypertrophy. The 37-day survival rate was 50%. To our knowledge, this study was the first to note the presence of complex vascular lesions, similar to those observed in patients with severe pulmonary arterial hypertension, in an isolated MCT model. (Arq Bras Cardiol. 2020; 115(3):480-490)

## Introduction

Pulmonary arterial hypertension (PAH) is a very severe clinical condition characterized by pulmonary vasoconstriction, *in situ* thrombosis and vascular remodeling.^1^ The progressive increase in pulmonary vascular resistance (PVR) causes compensatory right ventricular (RV) hypertrophy, leading to heart failure and premature death.^2^

In advanced PAH, endothelial proliferation and hypertrophy of vascular smooth muscle cause obstruction of the arterial lumen. In most cases, there are concentric intimal lesions and complex hypercellular lesions, known as plexiform lesions.^3^ The anatomical and pathological changes of PAH were first characterized by Heath & Edwards^4^in 1958. Later, Wagenvoort & Wagenvoort^5^described the sequence of vascular alterations in PAH and defined it as plexogenic pulmonary arteriopathy (PPA). These changes seem to reflect pulmonary artery pressure levels and, in a lesser extent, the time of PAH.^6^

Since tissue samples from human patients with diseases at an early stage are rarely available, an animal model that reproduces initial changes of pulmonary arteriopathy and its progression would be helpful to understand the complexity of PAH and find new therapeutic strategies.^7^

The animal model of monocrotaline (MCT)-induced PAH is one of the most commonly performed by researchers due to its technical simplicity, high reproducibility and low cost compared with other metods.^7^In addition, the method is able to mimic key aspects of PAH in humans, including vascular remodeling, smooth cell proliferation, endothelial dysfunction, increased expression of inflammatory cytokines and right ventricular failure.^1,8,9^ This model is based on the administration of one single dose of MCT (usually 60 mg/Kg), intraperitoneally or subcutaneously, leading to the development of PAH within 3-4 weeks thereafter.^7,10^

MCT is a pyrrolizidine alkaloid found in the stem, leaves and seeds of plants of the Crotalaria genus (mainly from the spectabilis, retusa and sigma species), which is a leguminous plant found mostly in tropical areas.^10^ MCT is metabolized by the cytochrome p450 in the liver, and transformed into its active form, the monocrotaline pyrrole, which has cardiopulmonary toxicity.^7,10^The use of MCT was first described more than 50 years ago by Kay et al.,^11^ who were the first to describe PAH caused by the ingestion of seeds of *Crotalaria spectabilis*in rats. Other reports had described pulmonary arteritis in rats fed with the seeds of the same plant,^12^ and identified pyrrolizidine alkaloids as the causative agent.^13^

Although many evidences suggest that MCT induces endothelial dysfunction at several levels, the model of PAH induced by MCT is predominantly characterized by hypertrophy of the middle layer. The absence of complex lesions seen in moderate-to-severe PAH may be an important limitation of this model.^7,14-16^

The present study aimed to reproduce the MCT-induced PAH model in Wistar rats to assess the severity of pulmonary arteriopathy by anatomopathological analysis of pulmonary, cardiovascular repercussions, clinical course and 37-day survival.

## Methods

The present experimental study followed the Guide for the Care and Use of Laboratory Animals recommendations and the ethical principles of the National Council on the Control of Animal Experiments (CONCEA). The study was approved by the ethics committee on animal experimentation of Mackenzie Evangelical School of Parana (CEUA/FEMPAR) (registration number 3433/2016).

Fifty Wistar (Rattus norvegicus) male rats, weighing 250-300 g were randomized (simple randomization) to one of the four groups: control group (CG) (n=10) – animals that received intraperitoneal injection of saline (0.9%, 1mL/Kg) at the beginning of the experiment (D0); MCT groups – animals that received intraperitoneal injection of MCT (60 mg/Kg)^17^ (Sigma-Aldrich, St. Louis, MO, USA), dissolved in saline (0.9%) on D0, and were assessed for the effects of MCT 15 days (G15, n=10), 30 days (G30, n=10) and 37 days (G37, n=10) thereafter.

On the 15^th^ (G15), 30^th^ (G30) and 37^th^ (G37 and CG) day of experiment, the animals were anesthetized with intraperitoneal 0.3 mg/Kg xylazine hydrochloride 2% (Xilazin^®^; Syntec, São Paulo, Brazil) and 100 mg/Kg ketamine 10% (Cetamin^®^; Syntec, São Paulo, Brazil),^17^ weighed, and sacrificed by exsanguination by cardiac puncture. Both lungs and the heart were removed and weighed using a portable balance (AD200; © Marte Científica, São Paulo, Brazil).

The organs were processed using conventional histological techniques. For each animal, lung tissue sections were placed on two glass slides, one containing three cross sections of the right pulmonary lobes, and the other containing one cross section and one longitudinal section of the left pulmonary lobe. The slides were stained with hematoxylin and eosin (H&E), Mallory trichrome and Weigert’s stain for elastic fibers.^14^ Heart tissue sections constituted of two cross sections, one at the mid-third of the ventricles and the other at the vessels of the base, stained with H&E.

The lung slides were analyzed histopathologically using a semiquantitative scoring system (0 – no changes; 1- mild; 2- moderate/severe) for vascular remodeling parameters and parenchymal changes, as described as follows: alveolar wall thickening, interstitial edema, alveolar exudate, middle layer hypertrophy, leukocyte infiltration and intimal/neointimal proliferation.

Lung and heart slides were digitized by a whole slide scanner (Axio Scan Z1, Zeiss, Jena, Germany) (40X) and the images analyzed using the ZEN software 2.3 (blue edition) (© Carl Zeiss Microscopy GmbH, 2011), which allows quantitative geometric measurements. Histological quantification of the RV wall thickness was measured at the central region of the RV wall, at 10x magnification and expressed in micrometers (µm). The area of the RV chamber was measured at 10x magnification and expressed in micrometers (µm^2^).

### Statistical analysis

The morphometric (continuous) variables were described in boxplots, which display the data in median and interquartile range. The normality assumption of the variables was assessed by the Kolmogorov-Smirnov test. The data did not show a normal distribution and then were analyzed and compared by the non-parametric Kruskal-Wallis test and the Simes and Hochberg multiple comparison method.

Sample size and allocation into the groups were conducted based on our experience in these animals and these experimental conditions, on the statistical viability of the experimental design, on medical literature and the norms established by the CONCEA and CEUA/FEMPAR. The Kolmogorov-Smirnov test indicated the need for eight animals per experimental group. Considering a mortality rate of 20% per group due to experimental problems, and of 50% per group from the fourth week on due to MCT toxicity, another two animals were added to the CG, G15 and G30 and twelve animals were added to the G37, with a final sample of 50 animals.

P values lower than 0.05 were considered statistically significant. Data were analyzed using the Action Stat software, version 3.5.152.34.

## Results

### Effects of MCT and Mortality

During the weeks of the experiment, animals of the MCT group gradually showed signs of pulmonary disease, such as anorexia, weight loss, irregular respiratory pattern, exertion intolerance and cyanosis of the extremities. These signs were more evident in the G30 and G37 groups, which were more irritated and sneezed more. All animals survived in the CG, G15 and G30, but the G37 showed a 50% mortality rate.

### Body weight and relative weight of the lungs and heart

As the time of MCT exposure increased, body weight of the animals decreased, as can be seen in Figure 1A. Thus, animals in the G37 showed a significant reduction in body weight, by nearly 11% from baseline weight, compared with CG and G30 (p<0.0001).

**Figure 1:** – Variation of body weight and relative weight of organs. Comparison between animals in the control group (CG), animals on day 15 (G15), on day 30 (G30) and on day 37 (G37). A, body weight variation, in grams, *p≤0.0001; B, relative cardiac weight (rHW), in g/100g of final weight (FW), *p<0.005 and **p≤0.0000; C - D, relative left and right lung weight (rLLW and rRLW), in g/100g of FW, *p≤0.0000.

Relative weight of the lungs was significantly higher in the MCT groups compared with the CG (p<0.0000) (Figures 1C and 1D). However, this increase did not show a linear pattern, with a slight fall in the G30. Relative weight of the hearts was significantly higher in the G30 and G37 (p<0,005) compared with controls (Figure 1B).

### Macroscopic findings

The lungs of the animals in G15 and G30 were macroscopically increased; some of the lungs did not show any findings on the surface, while others showed hyperemia and congestion. Most animals in the G37 showed severe pulmonary congestion, 30% with pulmonary petechiae and 20% with ferruginous bodies. At a macroscopic level, the right lung was more severely affected than the left lung. On sacrifice day, half of the animals in G37 presented serous fluid in pleural cavities and serohematic fluid in the pericardial cavity. The other organs did not show any macroscopic changes, except the liver, which was congested and with nutmeg aspect.

### Pulmonary histopathological findings

Photomicrographs of pulmonary tissue of the CG animals showed intact, normally aerated alveoli in most areas of the pulmonary parenchyma (Figures 2B and 2C), and arterioles with normal structure and size (Figures 2A, 3A, 3B and 4A).

**Figure 2:** – Photomicrographs of lung tissue in control group. Photomicrographs showing intact alveoli in most areas of pulmonary parenchyma (B and C) and arterioles with normal structures and dimensions (A). Hematoxylin-eosin staining; lens 20X (A), 5X (B) and 2X (C) objective lenses.

**Figure 3:** – Vascular and parenchymal histopathological findings of lung tissue. Photomicrographs of histological sections of lung tissue of animals of the control group (CG) and animals that received monocrotaline (MCT); A – F: cross sections showing pulmonary arterial remodeling in alveolar arterioles (Weigert’s stain; 20X objective lens); G – I: parenchymal changes in animals of the MCT groups (hematoxylin-eosin staining; 1X, 5X and 5X objective lenses, respectively); A and B alveolar arterioles with normal structure and dimensions; C – F, arterioles of groups 15, 30 and 37 days; C and D, muscularization and hypertrophy of the middle layer, with concentric neointimal proliferation; E and F, concentric, laminar neointimal lesions with important reduction of the vascular lumen and rupture of the external elastic membrane (E); G, peribronchial, perivascular, and hilar interstitial infiltrates; pulmonary parenchyma small areas of aerated alveoli associated with intense serofibrinous (H) and macrophagic (I) intra-alveolar exudation and sponge cells.

**Figure 4:** – Complex vascular lesions in lung tissue. Photomicrographs showing complex lesions in lung tissue samples of animals that received monocrotaline (MCT) (hematoxylin-eosin staining; lens 10X objective lens); A, alveolar arteriole in the control group with normal structure and dimensions; B – H, plexiform-like (“stalk-like”) lesions in cross sections; B – D, arterioles exhibiting “slit-like” channels (arrows); E – H, arterioles with “bud-like”, small mass of hyperchromatic and oval cells protruding from the arterial wall into the lumen (arrows); I – M, cross sections of plexiform lesions (animals on day 37, G37) showing a combination of multiple small capillary channels and narrow slits.

Histological analysis of the MCT groups revealed pulmonary parenchymal changes, such as alveolar exudate, alveolar wall thickening, interstitial edema and leukocyte infiltrate (Figure 3G). Three types of exudate were found in the alveolar lumen – serofibrinous (Figure 3H), hemorrhagic and macrophagic (Figure 3I). The pulmonary arteries and arterioles showed many forms of vascular remodeling, including muscularization and hypertrophy of the middle layer (Figures 3C and 3D), intimal proliferation, concentric neointimal thickening at several levels (Figures 3E and 3F) and complex hypercellular lesions.

In the muscularization process, the arteriole acquired a double elastic membrane and formation of new muscle between them. Proliferation of intimal cells formed a thickened intima, with no typical arrangement, affecting the entire circumference of the vessel.

Two morphologic patterns of complex hypercellular lesions were found. First, stalk-like/plexiform-like lesions, formed within the vessel lumen (Figures 4B-H); the body of the lesion was like a disorganized stalk-like mass comprising hyperchromatic and oval cells that appears to arise from the arterial wall and extend downstream into the lumen of the vessel. The cross section showed arterioles exhibiting many slit-like channels, separated by hyperchromatic core cells (Figures 4B-D). Also, there were “bud-like” small mass of cells protruding from the arterial wall into the lumen (Figures 4E-H).

The second pattern was plexiform lesions (Figures 4I-M), showing dilated arteries with capillary channels and slit-like channels within the lumen. These lesions had a focal distribution throughout the pulmonary tissue and were found almost exclusively in the G37, but not in all animals of the group.

In the G30 and G37, vascular and parenchymal lesions were more severe, particularly hypertrophy of the middle layer, intimal proliferation and alveolar exudate. However, the G37 showed a reduction in the leucocyte infiltrate compared with G15 and G30.

Results of the semiquantitative evaluation of pulmonary vasculature and parenchyma are illustrated in Figure 5.

**Figure 5:** – Progression of vascular remodeling parameters and parenchymal changes. Semiquantitative assessment (0 – no change; 1 – slight; 2 – moderate/severe).

### Histopathological and morphometric findings of the heart

The heart muscle also showed important anatomopathological changes. Figure 6 shows micrographs of cross and longitudinal sections of the right ventricle of animals of the CG and G37. There were intact nuclei arranged in the periphery and intact, but with increased diameter and thickness, fiber bundles in the G37 animals.

**Figure 6:** – Histopathological findings in cardiac tissue. Photomicrographs of cross (left) and longitudinal (right) sections of the right ventricle of animals in the control group (CG) and animals on day 37 (G37) (hematoxylin-eosin staining; lens 20X objective lens).

Right ventricular hypertrophy, clearly shown in Figure 7, was quantified by the measurement of RV wall thickness and its variation between the groups (Figure 8). RV wall thickness was not significantly different between CG and G15 and was significantly increased in G30/G37 (p<0.0000), almost twice the value in the CG.

**Figure 7:** – Progression of right ventricular hypertrophy and dilation. Photomicrographs of cross sections at the mid-third of the ventricles of animals in the control group (CG), animals on day 15 (G15), day 30 (G30) and day 37 (G37) (hematoxylin-eosin staining)

**Figure 8:** – Comparison of right ventricular wall thickness and right ventricular chamber area between the groups. Comparison of right ventricular wall thickness and right ventricular chamber area between the animals of the control group (CG), animals on day 15 (G15), on day 30 (G30) and on day 37 (G37), *p < 0.0000.

Another important finding was the significant dilatation of the RV chamber of the G30 and G37 (Figure 7), quantified by the measurement of the RV ventricular chamber area (p<0.0000) (Figure 8).

## Discussion

This was the first report to show the development of PPA following intraperitoneal administration of MCT (60mg/Kg) in rats.

Signs of pulmonary disease were gradually seen in the MCT group, in accordance with previous studies in which this animal model was reproduced.^9,18,19^ In most cases, dyspnea and weight loss are the first signs of pulmonary disease. Changes in blood pH secondary to an inefficiency of the lungs in maintaining a correct ventilation/perfusion ratio (which explains cyanosis in the extremities) contributes to anorexia and little weight gain. In the present study, cyanosis in the extremities of the animals could also be caused by peripheral vasoconstriction and low cardiac output. Exertional dyspnea increases metabolic expenditure, contributing to a low weight gain or weight loss. In fact, the most common sign of PAH is progressive dyspnea or fatigue resulting from low cardiac output, indicative of secondary RV failure.^20,21^

The increased relative lung weight and relative heart weight of the rats following MCT administration is well established in the literature.^9,17,18,22^ The increase in relative lung weight is caused by congestion, with reduced alveolar volume and increased extra-alveolar structures, especially alveolar exudate, alveolar wall thickening and interstitial edema. Analogously, the increase in relative heart weight may be secondary to increased mass of the hypertrophied right ventricle in MCT rats, which will be discussed below.

The active form of MCT was able to cause pulmonary vasculature and parenchyma changes, typical of PAH, in rats as young as 15 days. This finding corroborates the studies by Martins.^9^ Other authors, however, have found that significant changes in pulmonary arterial pressure, hypertrophy of the middle layer of pulmonary arterioles, and RV hypertrophy occur only 3-4 weeks after MCT administration.^18,23,24^

The endothelium exerts an important role in the etiopathogenesis of PAH, with active participation in tonus change and vascular remodeling, in addition to its involvement in vaso-occlusive events. Despite ample evidence for a correlation between MCT and endothelial barrier dysfunction, at several levels,^8,25,26^ according to many studies, this model is predominantly characterized by hypertrophy of the middle layer of pulmonary arterioles rather than exacerbation of endothelial proliferation, which is seen in PAH in humans.^3,17,27^ These findings contrast with histological analysis of the lung tissue samples in our study, since both hypertrophy of the middle layer and endothelial proliferation played a remarkable role in the development of pulmonary arteriopathy in the MCT animals, generating complex vascular lesions, referred as “plexiform” and “plexiform-like” lesions.

Although the plexiform lesions found in severe human PAH are difficult to be reproduced in animal models, for the first time, an MCT model alone showed the presence of these lesions. Plexiform lesions are characterized by a disorganized, glomeruloid-like vascular lesions, comprising hyperchromatic and oval cells.^3-5^ These features, despite unique, are uncertain, probably because of the dynamic behavior of the lesion and, more importantly, it is not known how the lesion is formed. It is a consensus that plexiform lesions are complex, angioproliferative lesions, composed of channels lined up by endothelial cells separated by core cells.^28,29^ These complex lesions described in our study are relatively similar to the morphology of plexiform lesions in severe PAH in humans.

As recently suggested by Tuder,^28^ in a review of arteriopathy of PAH in humans, plexiform-like lesions can represent the initial stage of the intraluminal pattern of the plexiform lesion. As the name suggests, the complexity of these lesions resembles that of typical plexiform lesions, but with different morphology. These lesions, identified in our study, are formed within the vascular lumen, with the body of the lesion resembling a disorganized stalk-like mass, composed of hyperchromatic and oval cells that appeared to arise from the arterial wall and extend into the lumen of the vessel, showing “slit-like” channels and “bud-like” structures.^30^

Many combined models of PAH described in the literature have shown that hemodynamic changes and changes in oxygen saturation levels in animals subjected to inoculation of toxic substances can intensify the effects of these compounds on pulmonary arteries and consequently lead to the development of complex vascular lesions. White et al.^31^ reported severe PAH in young pneumonectomized rats following MCT inoculation. Coste et al.^32^ and Morimatsu et al.^33^ demonstrated aggravation of PAH by MCT administration in rats subjected to chronic hypoxia. Also, Abe et al.^30^ reported the development of PAH and severe pulmonary arteriopathy following injection of the vascular endothelial growth factor receptor blocker Sugen 5416 and chronic hypoxia. The neo-intimal concentric laminar and nonlaminar lesions, observed in our study, are similar to those reported in severe human PAH^34^ and the experimental models of PAH mentioned above.

Wagenvoort & Wagenvoort^5,35^ were the first to describe the sequence of vascular alterations in PAH and one of the pioneers in evaluating the degree of reversibility of the lesions, and their studies are still valid today. In our study, muscularization, hypertrophy of the middle layer and intimal/neointimal proliferation were the first abnormalities in this process and, thus, potentially reversible in the anatomical-pathological point of view. However, complex vascular lesions, more common in the G37 of our study, can be considered generally irreversible, resulting in more severe hemodynamic changes and early mortality, as indeed observed.^31^

The detection of plexiform lesions in our study and may be related to the prolonged observation time (37 days) and the relatively more comprehensive anatomopathological analysis of the lung samples. The development of complex vascular lesions in PAH is time-dependent, *i.e*, the longer the exposure time of the animals to MCT, the higher the risk of progression to more complex lesions. Besides, the plexiform lesions in the pulmonary parenchyma are focal, and thereby dependent on the extent of the histological analysis.

With the PAH progression, the increase in pulmonary vascular resistance causes a secondary RV hypertrophy due to RV overload.^36^ In our study, RV hypertrophy was found in the animals after 30 days of MCT exposure.^9,36,37^ Myocardial hypertrophy is not necessarily related to greater cardiac contraction and progresses to dilation of the right chambers and *cor pulmonale*.^9,17^ Signs of heart failure such as pleural effusion, ascites and hepatic congestion, were detected in animals of the G37, in accordance with studies cited earlier.

RV wall thickness was used as marker of RV hypertrophy, although many studies have used the ratio RV weight to left ventricular + interventricular septum weight instead.^17^ Both methods allowed to demonstrate the development of considerable RV hypertrophy in MCT animals. Also, RV hypertrophy was evidenced by an increase in the thickness and diameter of cardiomyocytes, with preservation of fiber integrity, similar to what was described by Martins,^9^ Cabrini,^18^ Nogueira-Ferreira et al.^38^ and Pacagnelli et al.^39^ Finally, the progressive dilation of the right chambers corroborates the findings of Martins,^9^ who described higher dimensions in the right heart compared with the left side of the heart.

Despite similarities, this model does not precisely mimic human PAH. The main reason is that it is not known how complex angioproliferative lesions are formed and whether they actually reproduce the pathophysiological aspects of the lesions in humans.

## Conclusion

The experiment showed that an intraperitoneal dose of MCT (60mg/Kg) was able to cause moderate-to-severe pulmonary arteriopathy, with muscularization of arterioles, hypertrophy of the middle layer and neointimal formation. The model successfully reproduced several structural changes in lung parenchyma and pulmonary arterioles, as well as RV hypertrophy secondary to increased pulmonary vascular resistance. To our knowledge, this is the first study to detect complex lesions, mainly plexiform, similar to those found in severe PAH, in an isolated MCT model.
